# Cost-neutral reduction of infection risk in picker-to-parts warehousing systems

**DOI:** 10.1007/s00291-022-00695-8

**Published:** 2022-11-23

**Authors:** Maximilian Löffler, Michael Schneider, Ivan Žulj

**Affiliations:** 1 Deutsche Post Chair – Optimization of Distribution Networks, RWTH Aachen University, Aachen, Germany; 2 Department of Procurement and Production, University of Hohenheim, Stuttgart, Germany

**Keywords:** Routing, Iterated local search, Picker routing, COVID-19, Pandemics

## Abstract

The rapid and severe outbreak of COVID-19 caused by SARS-CoV-2 has heavily impacted warehouse operations around the world. In particular, picker-to-parts warehousing systems, in which human pickers collect requested items by moving from picking location to picking location, are very susceptible to the spread of infection among pickers because the latter generally work close to each other. This paper aims to mitigate the risk of infection in manual order picking. Given multiple pickers, each associated with a given sequence of picking tours for collecting the items specified by a picking order, we aim to execute the tours in a way that minimizes the time pickers simultaneously spend in the same picking aisles, but without changing the distance traveled by the pickers. To achieve this, we exploit the degrees of freedom induced by the fact that picking tours contain cycles which can be traversed in both directions, i.e., at the entry to each of these cycles, the decision makers can decide between the two possible directions. We formulate the resulting picking tour execution problem as a mixed integer program and propose an efficient iterated local search heuristic to solve it. In extensive numerical studies, we show that an average reduction of 50% of the total temporal overlap between pickers can be achieved compared to randomly executing the picking tours. Moreover, we compare our approach to a zone picking approach, in which infection risk between pickers can be almost eliminated. However, compared to our approach, the results show that the zone picking approach increases the makespan by up to 1066%.

## Introduction

Warehousing is an essential component of many supply chains (see, e.g., Boysen et al. 2021) and has a strong influence on on-time deliveries and customer satisfaction. High cost pressure, steadily increasing global retail sales volumes (Statista 2019), and next- or same-day deliveries force warehouse managers to improve the performance of their warehousing processes to meet the customer expectations for fast delivery and to gain advantages over competitors (see, e.g., Weidinger 2018; Boysen et al. 2019).

The most labor-intensive warehousing activity is order picking, which is the process of retrieving items from their storage locations to fulfill customer orders. Studies indicate that a large share of all order picking systems in Western Europe follow the traditional picker-to-parts warehousing setup, in which pickers move through the warehouse to retrieve the requested items from their storage locations (Napolitano 2012). The major drawback of such systems is the large fraction of unproductive picker walking time (de Koster et al. 2007; Tompkins et al. 2010). While there are technologies to automate order picking (see, e.g., Azadeh et al. 2019), warehouse managers rely on manual order picking because human pickers are flexible and can adapt to changes in real time compared to automated systems (Grosse et al. 2014).

The rapid spread of SARS-CoV-2 forces warehouse managers to reduce the risk of spreading the virus to avoid a complete stop of the workflow due to infected pickers. Because SARS-CoV-2 is mainly spread person-to-person through close contact, and pickers work close to each other, the risk of virus transmission in manual order picking is very high. As a response to COVID-19, warehouse managers initially aimed at maintaining physical distance between pickers by restricting the number of pickers allowed in a picking area at one time or by implementing one-way picking aisles to avoid congestion or picker blocking (DC Velocity 2020). Because such safety measures make it hard to meet pre-pandemic picking performance due to longer picking routes, Amazon, for example, launched a social distancing monitor based on artificial intelligence, which visually alerts pickers when physical distance is not maintained (Amazon 2020). Softeon, a global supply chain software vendor, offers a software that alerts a picker if the picker’s next pick is in a picking aisle which is already occupied by another picker (DC Velocity 2020).

**Fig. 1:**
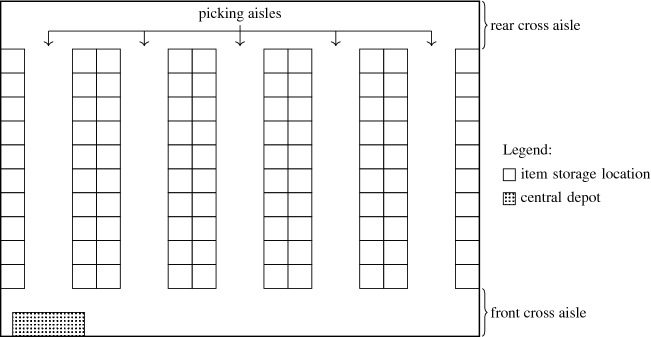
An example of a rectangular single-block warehouse layout

This study aims at mitigating the risk of becoming infected or spreading SARS-CoV-2 (or other infectious diseases) in manual order picking by minimizing the time pickers simultaneously spend in the same picking aisles, which supports maintaining social distancing practices. We assume that the risk of infection is proportional to the time that pickers spend in close proximity to each other, which is in accordance with the current state of knowledge (see, e.g., World Health Organization 2020). We consider a rectangular single-block warehouse layout (see Fig. 1) with a central depot and with parallel picking aisles that are connected by a cross aisle at the front and at the rear of the picking aisles. Items are stored in storage locations arranged along both sides of the picking aisles.

**Fig. 2:**
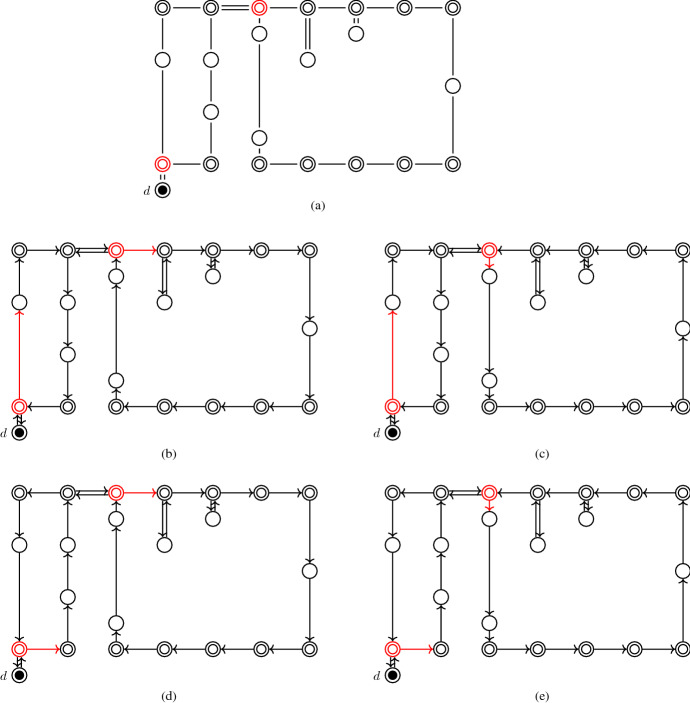
Example of different execution possibilities (b)–(e) of the picking tour given in (a)

We assume that each picker is given a sequence, in which the picking orders are to be executed. A picking order comprises a single or multiple customer orders. For each picking order, the picking tour along the storage locations is given. A common feature of picking tours is that they contain cycles which can be traversed in both directions, i.e., the decision maker can choose between the two possible directions at the entry to each of these cycles. Figure 2 gives an example of different execution possibilities (see Fig. 2(b)–(e)) of a picking tour (see Fig. 2(a)). The double circle marked by *d* represents the central depot, the other double circles indicate the front/rear locations of each picking aisle, and the standard circles denote the picking positions. The picking tour is represented by the solid lines. The front/rear end locations of picking aisles at which a degree of freedom occurs are marked red. Here, the picker can decide whether to enter the respective picking aisle (if so, we indicate this by a red vertical arc) or to proceed along the front/rear cross aisle (if so, we indicate this by a red horizontal arc). For each of the four depicted execution possibilities, the time periods in which the picker occupies a certain picking aisle differ. Consequently, to reduce virus transmission among pickers, we seek to execute the tours such that the time that pickers spend simultaneously in the same picking aisles is minimized. Note that the decision on how the picking tours are executed does not affect the distance traveled by the pickers.

The risk of infection in the cross aisles is not considered because cross aisles are generally significantly wider than the picking aisles, and thus, wide enough to allow pickers to pass each other while maintaining a social distance. Moreover, the risk of virus transmission via goods is assumed to be negligible for the following reason: when a picker moves to the storage location of a requested item, she^1^ does not touch the surrounding items. The risk of infection via goods can be further decreased by regularly disinfecting hands.

**Fig. 3:**
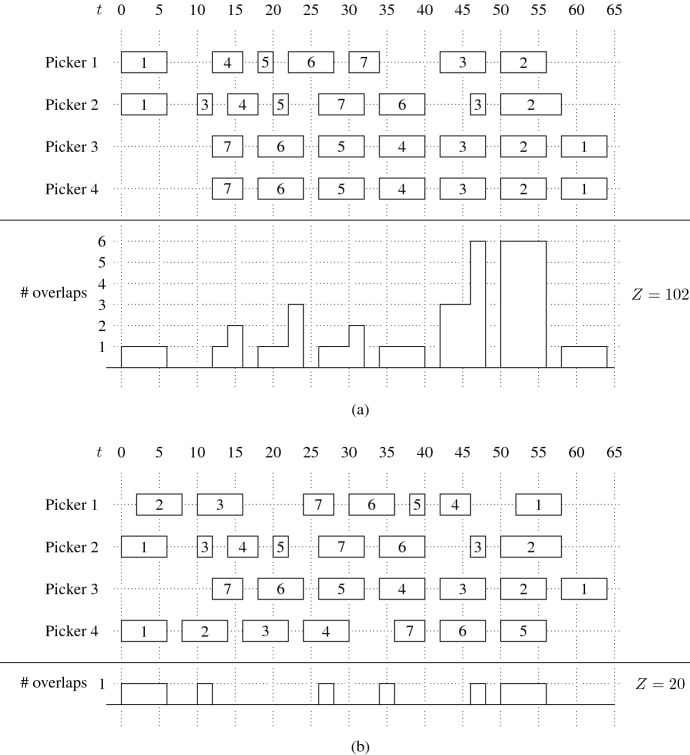
Total temporal overlap between pickers (*Z*) for different picking tours

In Fig. 3, we show the impact of different executions of picking tours on the total time pickers spend together in the same picking aisles. To this end, we consider an instance with four picking tours, each associated with one of four pickers. In the upper part of Fig. 3(a), the picking tours are illustrated by the sequence in which the picking aisles are visited by the respective picker. The number in a rectangle indicates the picking aisle, and the length of a rectangle represents the time period the respective picker spends in this picking aisle. For example, picker 1 starts in picking aisle 1 (from *t* $=$ 0 to *t* $=$ 6) and from there proceeds to picking aisles 4 (from *t* $=$ 12 to *t* $=$ 16), 5 (from *t* $=$ 18 to *t* $=$ 20), 6 (from *t* $=$ 22 to *t* $=$ 28), and so on. In the lower part of Fig. 3(a), we report the time that the pickers simultaneously spend in the same picking aisles resulting from the given execution of the picking tours. We assume that in the case in which *n* pickers occupy a picking aisle at a point in time, 0.5 $\cdot$ *n* $\cdot$ $(n-1)$ overlaps occur. Executing the picking tours given in Fig. 3(a) leads to a total temporal overlap of *Z* $=$ 102. Modifying the execution of the picking tours of pickers 1 and 4 as shown in the upper part of Fig. 3(b) leads to a reduction in the temporal overlap by approximately 80%. The example also shows that the length of the rectangles in Fig. 3(a) is the same as in Fig. 3(b), i.e., the distance traveled (or time required) by each picker is not affected.

To the best of our knowledge, the resulting picking tour execution problem (PTEP) constitutes a novel setting, which has not been studied in the literature so far but is of high relevance in real-world warehouses. The contributions of our paper are the following:

- We formally describe the PTEP as a mixed integer program and propose an efficient iterated local search heuristic (ILS) to provide solutions for large-sized problem instances.
- In extensive numerical studies, we test the performance of our PTEP model using the optimization software Gurobi. Our formulation is able to solve small and medium instances but is not able to consistently solve large instances within a given time limit. Moreover, we compare the performance of ILS to those of Gurobi solving PTEP. The results clearly demonstrate the ability of ILS to find high-quality solutions within reasonable runtimes.
- Additional experiments show that reductions between 17% and 100% of the total temporal overlap between pickers can be achieved compared to randomly executing the picking tours without increasing the distance traveled by pickers.
- To provide managerial insights, we compare our PTEP to a zone picking approach, in which the risk of infection spread is almost eliminated. The results show that the zone picking approach strongly increases the makespan compared to our approach.

The remainder of the paper is structured as follows: In Sect. 2, we discuss the related literature. In Sect. 3, we introduce the PTEP and present a mixed integer formulation. Our ILS is detailed in Sect. 4. Section 5 is devoted to the numerical studies. In Sect. 6, we compare the PTEP to a zone picking approach, followed by a conclusion in Sect. 7.

## Literature review

Because traveling is considered the most time-consuming warehousing activity, research mainly focuses on reducing the average distance traveled (or time required) by pickers for collecting the items of a given set of picking orders. Travel distance depends on the design of the following four planning problems: warehouse layout (configuration of the warehouse, including the number of blocks and the number, length, and width of the picking aisles and cross aisles in each block), picker routing (determining the picking tour through the warehouse and the retrieval sequence of the items), order batching (grouping or splitting of customer orders), and storage assignment (assignment of items to storage locations).

The PTEP is closely related to picker routing problems (PRPs), which, in general, aim at determining a cost-minimal picking tour along the storage locations defined by a picking order (see, e.g., Ratliff and Rosenthal 1983). The most well-studied PRP in the literature is the standard single picker routing problem (standard SPRP), which can be defined as follows: Given a single-block parallel-aisle warehouse with a central depot and dedicated storage, i.e., each item is available from one storage location, the standard SPRP seeks the cost-minimal picking tour for collecting the items defined by a picking order. For an extensive review on PRPs, we refer the reader to Masae et al. (2019). Contrary to PRPs, in our PTEP, the picking tour is already given as input, and it can be generated by an arbitrary solution method for the PRP. Moreover, in the PTEP, picking tours are executed such that the temporal overlap between pickers is minimized, whereas previous research on PRPs has mainly concentrated on determining picking tours such that the distance traveled (or time required) by pickers is minimized.

The risk of infection spread in warehousing operations is only considered in Ardjmand et al. (2021). The authors study an order batching problem which aims at grouping customer orders into (larger) picking orders such that the total order picking time, the makespan, and the time in which pickers are picking closer than at a specified physical distance are minimized. To solve the resulting problem, they propose three multi-objective metaheuristics. Ardjmand et al. (2021) do not aim to reduce the risk of infection by optimizing the routing of pickers but assume that pickers are fixedly routed according to an S-shape routing policy.

Another closely related problem concerns the blocking of pickers. The studies that exist on picker blocking consider additional travel distance (or time) that occurs when, for example, pickers meet and passing one another is not possible due to narrow picking aisles. Gue et al. (2006) investigate the effect of pick density on picker blocking in warehousing systems with high space utilization, in which pickers move in an S-shape fashion along unidirectional picking aisles, and passing by other pickers is not allowed. To avoid blocking, the authors discuss different routing methods, e.g., forcing a blocked picker to leave the picking aisle and proceed along a picker-free picking aisle to continue order picking. Pan and Shih (2008) and Parikh and Meller (2009) investigate a multiple-picker setting with congestion considerations from a queuing theory perspective: Pan and Shih (2008) present a throughput model for a picker-to-parts warehouse involving multiple pickers and narrow picking aisles, which simultaneously considers the total travel time and the congestion effect. A picker is routed according to the S-shape routing policy, and in case that an aisle is already occupied by another picker, she has to wait in a buffer zone until the other picker leaves the picking aisle. Parikh and Meller (2009) develop analytical models to estimate picker idle time in a wide-aisle distribution center, in which picker blocking occurs if a picker blocks access to a picking position of another picker. Zhang et al. (2009) consider workflow congestion in the context of material handling equipment interruptions in a manufacturing or warehousing facility. The authors combine a probabilistic and physics-based model to evaluate the expected link travel time when interruptions occur. Two heuristic algorithms are developed to solve the combined optimization model. Hong et al. (2012) introduce an integrated batching and batch sequencing problem for a narrow-aisle order picking system that aims at minimizing the sum of travel time, pick time, and congestion delays. To address realistically sized instances, the authors present a simulated annealing heuristic. Chen et al. (2013, 2014) consider a warehousing system with narrow picking aisles, in which picker congestion has to be avoided. Chen et al. (2013) propose a routing algorithm based on ant colony optimization for two pickers and Chen et al. (2014) for multiple pickers. Schrotenboer et al. (2017) develop a genetic algorithm to evaluate the tradeoff between delays caused by so-called picker interactions and the time required to avoid an interaction. An interaction delay occurs, for example, when a picker blocks access to a storage location from which another picker needs to collect an item. To conclude, methods on picker blocking cannot be used to solve the PTEP because picker blocking problems assume that passing one another is not possible.

## The picking tour execution problem

In this section, we provide a detailed description of the PTEP (see Sect. 3.1) and present a mathematical formulation (see Sect. 3.2).

### Problem description

**Fig. 4:**
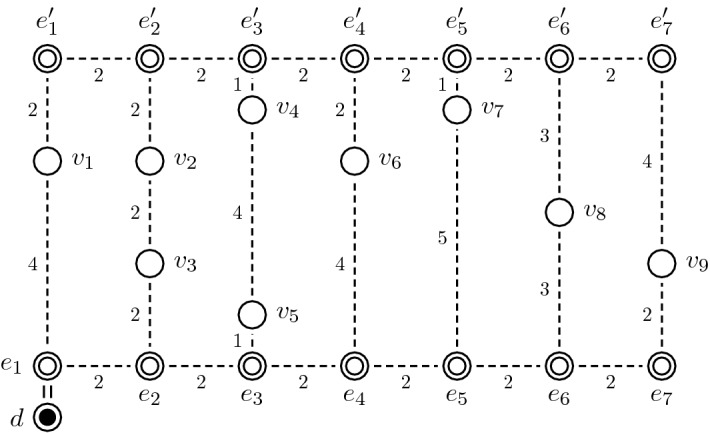
An example of a warehouse graph with |*A*| $=$ 7 picking aisles and |*I*| $=$ 9 picking positions

The PTEP considers a rectangular single-block warehouse with parallel picking aisles (see Fig. 1). Let *A* denote the ordered set of picking aisles, which are numbered in ascending order from the leftmost to the rightmost picking aisle. We use a weighted graph $G = (V, E)$ to describe a picking order within a warehouse. Set *V* indicates the vertices including the front (rear) locations $e_a$ ($e^\prime _a$) of each picking aisle *a* $\in$ *A*, the depot *d*, and the picking positions $v_i$ of each requested item *i* $\in$ *I*, where *I* denotes the set of requested items of the picking order. Set *E* consists of an unlimited number of parallel edges between every pair of adjacent vertices. The weight of each edge represents the travel distance (or time) between two adjacent vertices. An example of a warehouse graph with |*A*| $=$ 7 picking aisles and |*I*| $=$ 9 picking positions is given in Fig. 4. For simplicity, the figure represents the parallel edges connecting adjacent vertices by a single dashed edge.

The items of a given set of picking orders have to be collected. Let $K$ denote the set of pickers, where each picker *k* $\in$ *K* is associated with an ordered set $O^k$ of so-called tour subgraphs. A tour subgraph *o* is a subgraph of *G* from which a directed picking tour can be constructed such that every arc of the directed picking tour corresponds to exactly one edge in *o*. Note that the PTEP works independent of the underlying warehouse layout and routing policy because any routing policy used in any warehouse layout can be described such that the output is a tour subgraph, which is used as input to the PTEP and ILS. In Fig. 2(a), a tour subgraph returned by the optimal routing policy is depicted, and in Fig. 2(b)–(e), we show the four directed picking tours that can be constructed from the tour subgraph in Fig. 2(a). Theorem 1 states the formal properties of a tour subgraph and has been proved by Ratliff and Rosenthal (1983).

#### **Theorem 1**

(Properties of a tour subgraph) *A tour subgraph has the following properties*:

1. *Vertices* $v_i$ *and vertex* *d* *have nonzero even degree in* *o*.
2. *Excluding vertices with zero degree*, *o* *is connected*.
3. *Every vertex in* *o* *has even or zero degree*.

The PTEP aims to construct directed picking tours from given tour subgraphs such that the total temporal overlap between pickers is minimized. Therefore, we describe the procedure for constructing directed picking tours (see Theorem 2, proved by Ratliff and Rosenthal (1983)), and we show that picking tours generally contain cycles which can be traversed in both directions.

#### **Theorem 2**

(Procedure for constructing directed picking tours) *Given a valid tour subgraph, the directed picking tour can be constructed as follows*:

1. *Start the tour at the depot vertex* *d*.
2. *If there is a pair of unused parallel arcs in the tour subgraph incident to the current vertex, use an arbitrary one of them to get to the next vertex. Continue with* *step* 2.
3. *If there are any unused single arcs in the tour subgraph (i.e., not one of a pair of parallel arcs), use an arbitrary one of them to get to the next vertex. Continue with* *step* 2.
4. *If there is a pair of parallel arcs in the tour subgraph with one arc used and one arc still unused, use the unused arc to get to the next vertex. Continue with* *step* 2.
5. *Stop. The directed picking tour is complete*.

**Fig. 5:**
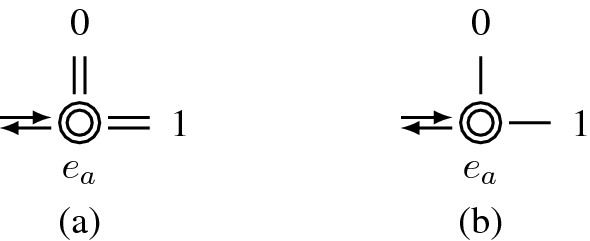
Degrees of freedom resulting from Theorem 2

In Theorem 2, a degree of freedom occurs in steps 2 and 3, respectively, i.e., each of these steps identifies a vertex associated with a degree of freedom. Figure 5 shows the two edge configurations for which degrees of freedom occur in a single-block parallel-aisle warehouse layout. For both configurations, there are two ways for selecting the next arc, and thus, at the entry $e_a$ to the respective cycle, the decision maker can decide between the two possible directions, represented by 0 and 1. Note that these degrees of freedom only occur at the front or rear end of a picking aisle.

In Fig. 5(a), the picker reaches vertex $e_a$ via the upper arc. At $e_a$, the picker can decide which of the edge pairs marked 0 or 1 she traverses first. Note that the picker traverses both edges of a parallel edge pair before she traverses the edges of the other edge pair. Such a configuration occurs in tour subgraphs in which the picking aisles are entered and left from the same cross aisle. In Fig. 5(b), the picker reaches vertex $e_a$ via the upper arc. At $e_a$, both of the edges belong to the same cycle. Here, the picker returns to vertex $e_a$ via the unselected edge, e.g., if edge 0 is selected first, she returns to vertex $e_a$ via edge 1.

As described, the picker’s decision influences the time periods in which she occupies a certain picking aisle without changing the distance traveled by the picker. We use this observation to minimize the time that pickers simultaneously work in the same picking aisles.

### Mathematical model formulation

This section introduces the notation used in the PTEP model and presents a mathematical model formulation for the PTEP.

Set $\Pi ^k_o$ contains all possibilities for executing tour subgraph $o$ $\in$ $O^k$ by picker *k* $\in$ *K*, where $\pi$ $\in$ $\Pi ^k_o$. Binary variable $\sigma _\pi$ denotes whether execution possibility $\pi$ $\in$ $\Pi ^k_o$ is selected ($\sigma _\pi$ $=$ 1) or not ($\sigma _\pi$ $=$ 0). We assume discrete points in time *t* $\in$ *T*. Because each execution possibility $\pi$ $\in$ $\Pi ^k_o$ represents a picking tour, the points in time at which picker *k* $\in$ *K* is in picking aisle $a$ $\in$ *A* executing tour subgraph *o* $\in$ $O^k$ can be determined before solving the PTEP model and are represented by set $T_{a\pi }$, where $T_{a\pi }$ $\subseteq$ *T*.

Finally, binary variable $x^n_{at}$ indicates whether *n* $\in$ *N* pickers are in picking aisle $a$ $\in$ *A* at time *t* $\in$ *T* ($x^n_{at}$ $=$ 1) or not ($x^n_{at}$ $=$ 0), where *N* is the set of possible numbers of pickers that simultaneously occupy a picking aisle.

Using the notation summarized in Table 1, the PTEP can be formulated as the following mixed integer program.

**Table 1:** Overview of the notation used in the PTEP model

*Sets*	
*A*	Ordered set of picking aisles (index: *a*)
*K*	Set of pickers (index: *k*)
*N*	Set of possible numbers of pickers that simultaneously occupy a picking aisle (index: *n*)
$O^{k}$	Ordered set of tour subgraphs associated with picker *k* $\in$ *K* (index: *o*)
*T*	Set of discrete points in time (index: *t*)
$T_{a\pi }$	Set of discrete points in time at which a picker is in picking aisle $a$ $\in$ *A* if execution possibility $\pi$ $\in$ $\Pi ^k_o$ is selected, where $T_{a\pi }$ $\subseteq$ *T* (index: *t*)
$\Pi ^k_o$	Set of all possibilities for executing tour subgraph $o$ $\in$ $O^k$ of picker *k* $\in$ *K* (index: $\pi$)
*Binary decision variables*	
$x^{n}_{at}$	1, if *n* pickers are in picking aisle $a$ $\in$ *A* at time *t* $\in$ *T*; 0, otherwise
$\sigma _\pi$	1, if execution possibility $\pi$ $\in$ $\Pi ^k_o$ is selected; 0, otherwise

1 $$\begin{aligned} \text {min }Z&\,=\,\sum _{a \in A} \sum _{t \in T} \sum _{n \in N:n\ge 2} \frac{n\cdot {}(n-1)}{2}\cdot {}x^n_{at} \end{aligned}$$

subject to

2 $$\begin{aligned} \sum _{n \in N:n\ge 2} (n - 1)\cdot {}x^n_{at}+1&\ge \sum _{k\in K} \sum _{o\in O^k} \sum _{\pi \in \Pi ^k_o\,:\,t \in T_{a\pi }} \sigma _{\pi }&\forall t \in T;\, a \in A \end{aligned}$$

3 $$\begin{aligned} \sum _{n \in N:n\ge 2} x^n_{at}&\le 1&\forall t \in T; \,a \in A \end{aligned}$$

4 $$\begin{aligned} \sum _{\pi \in \Pi ^k_o} \sigma _{\pi }&= 1&\forall k\in K; \,o\in O^k \end{aligned}$$

5 $$\begin{aligned} x^n_{at}&\in \{0, 1\}&\forall n \in N:n\ge 2;\,t \in T; \,a \in A \end{aligned}$$

6 $$\begin{aligned} \sigma _{\pi }&\in \{0, 1\}&\forall k\in K; \,o\in O^k;\,\pi \in \Pi ^k_o \end{aligned}$$

Objective function (1) minimizes the total temporal overlap between pickers. Constraints (2) ensure that binary variable $x^n_{at}$ is correctly defined by linking $x^n_{at}$ (left side) to the number of pickers at each point in time *t* $\in$ *T* and in each picking aisle $a$ $\in$ *A* (right side). To determine the number of pickers, we sum over all selected decision variables $\sigma _\pi$ for which a picker occupies picking aisle $a$ $\in$ *A* at point in time *t* $\in$ *T*. Constraints (3) ensure that the binary decision variable $x^n_{at}$ is chosen at most once for each picking aisle $a$ $\in$ *A* and point in time *t* $\in$ *T*, i.e., only *n* pickers (or none) can simultaneously occupy a picking aisle. Constraints (4) guarantee that exactly one decision variable $\sigma _\pi$ is selected for each tour subgraph *o* $\in$ $O^{k}$ assigned to picker *k* $\in$ *K*, i.e., exactly one directed picking tour is executed from the execution possibilities of the respective tour subgraph. Finally, the binary decision variables are defined in constraints (5) and (6), respectively.

## An iterated local search for the picking tour execution problem

We define binary variable $\pi ^k_j$ $\in$ $\{0, 1\}$, where index *j* $=$ $1,\ldots ,n^{k}_{o}$ denotes the *j*-th degree of freedom of tour subgraph *o* $\in$ $O^k$ of picker *k* $\in$ *K*. Index *j* is defined in ascending order based on its occurrence in the tour subgraph from left to right. Note that the *j*-th degree of freedom is associated with the entry at $e_a$ (or $e^\prime _a$) to a cycle at which picker *k* $\in$ *K* can decide whether to traverse picking aisle $a$ $\in$ *A* that is adjacent to vertex $e_a$ (or $e^\prime _a$) or to proceed along the front (or rear) cross aisle. Binary variable $\pi ^k_j$ takes value 0 if picker *k* $\in$ *K* enters picking aisle $a$ $\in$ *A* that is adjacent to vertex $e_a$ (or $e^\prime _a$) associated with the *j*-th degree of freedom, and 0 otherwise. Note that there are $|\Pi ^k_o|$ $=$ $2^{n^{k}_{o}}$ possibilities for executing tour subgraph $o$ $\in$ $O^k$ of picker *k* $\in$ *K*.

**Fig. 6:**
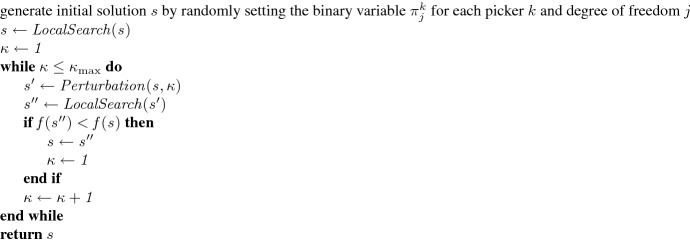
Overview of the iterated local search algorithm

In Fig. 6, a pseudocode overview of the basic ILS framework is given, which can be described as follows: First, the initial solution is determined by randomly setting the binary variable $\pi ^k_j$ for each picker *k* and degree of freedom *j*, i.e., we randomly define whether the picker first traverses the picking aisle associated with *j* (i.e., $\pi ^k_j$ $=$ $0{}$) or proceeds along the respective cross aisle (i.e., $\pi ^k_j$ $=$ $1{}$). Second, the local search is applied to an initial solution *s*. The neighborhood $s^\prime$ used in the local search step is defined by all possible inversions of one of the binary decision variables $\pi ^k_j$. Third, an incumbent solution $s^\prime$ is generated by perturbating *s*. The strength of the perturbation is defined dynamically as follows: At iteration $\kappa$, $\frac{\kappa }{\kappa _{\max }}\sum _{k\in K}\sum _{o\in O^k} |\Pi ^{k}_{o}|$ randomly chosen decision variables are inverted, where $\kappa _{\max }$ denotes the number of iterations without improving the best found solution. Fourth, the local search is applied to $s^\prime$. The generated solution $s^{\prime \prime }$ is accepted as the new current solution if it is better than the current solution *s*, i.e., if $f(s^{\prime \prime })<f(s)$, where $f(\cdot )$ denotes the total temporal overlap between pickers. To achieve a good tradeoff between the solution quality and the runtime of our algorithm, the ILS terminates after $\kappa _{\max }$ $=$ 10. Higher numbers of iterations slightly improve the average solution quality but significantly increase the runtimes.

## Numerical studies

This section presents the numerical studies (i) to assess the performance of our PTEP formulation using Gurobi, (ii) to compare the performance of ILS to that of Gurobi solving PTEP, and (iii) to give managerial insights with respect to the potential avoidance of temporal overlaps between pickers. In Sect. 5.1, we describe the test instances, and in Sect. 5.2, we present the results of our computational experiments.

### Test instances

Our experiments are based on the instances of Henn and Wäscher (2012), originally designed for the order batching problem. The instances consider a single-block parallel-aisle warehouse with ten parallel picking aisles, each containing 90 different items (45 on the left and 45 on the right). The depot is located below the entry of the leftmost picking aisle in the front cross aisle. The physical dimensions of the warehouse are as follows: An order picker has to cover 1 length unit (LU) (or 1 time unit) to get from the depot to the front cross aisle. The distance between the front cross aisle and the first storage location of a picking aisle is 1 LU, and the distance between two neighboring picking aisles is 5 LUs. The authors assume two different demand scenarios, i.e., uniformly distributed demand (UDD) and class-based demand (CBD). For UDD, items are randomly assigned to storage locations. For CBD, they define three classes with high (A), medium (B), and low (C) demand frequencies. Class A contains 10% of the items that account for 52% of the demand, class B 30% of the items that account for 36% of the demand, and class C 60% of the items that account for 12% of the demand. Items of class A are stored in picking aisle *a* $=$ 1, items of class B in picking aisles *a* $=$ 2 to *a* $=$ 4, and items of class C in picking aisles *a* $=$ 5 to *a* $=$ 10. The authors consider groups of 40 instances, which are identified by the demand scenario and the number of picking orders (20, 40, 60, 80, and 100). The number of items per picking order is randomly drawn from the interval [2, 25].

In our experiments, we consider |*K*| $=$ $\left\{ 2, 5, 10, 20, 40\right\}$ pickers and $|O^k|$ $=$ $\left\{ 2, 5, 10, 20\right\}$ tour subgraphs per picker *k* $\in$ $K$. To use the instances of Henn and Wäscher (2012) for our experiments, we make the following adjustments: We select the first 2, 5, 10, or 20 picking orders from an instance of Henn and Wäscher (2012), who originally assume 20, 40, 60, 80, or 100 picking orders, to generate $|O^k|$ $=$ $\left\{ 2, 5, 10, 20\right\}$ tour subgraphs. Moreover, their instances consider a single picker and thus cannot be used directly for our experiments. However, given that 40 instances were generated per instance group, we select the first 2, 5, 10, 20, or 40 instances of a group, and we use, e.g., the two selected instances for the case of |*K*| $=$ 2 pickers, one for each of the pickers, the 5 selected for |*K*| $=$ 5 pickers, and so on.

**Fig. 7:**
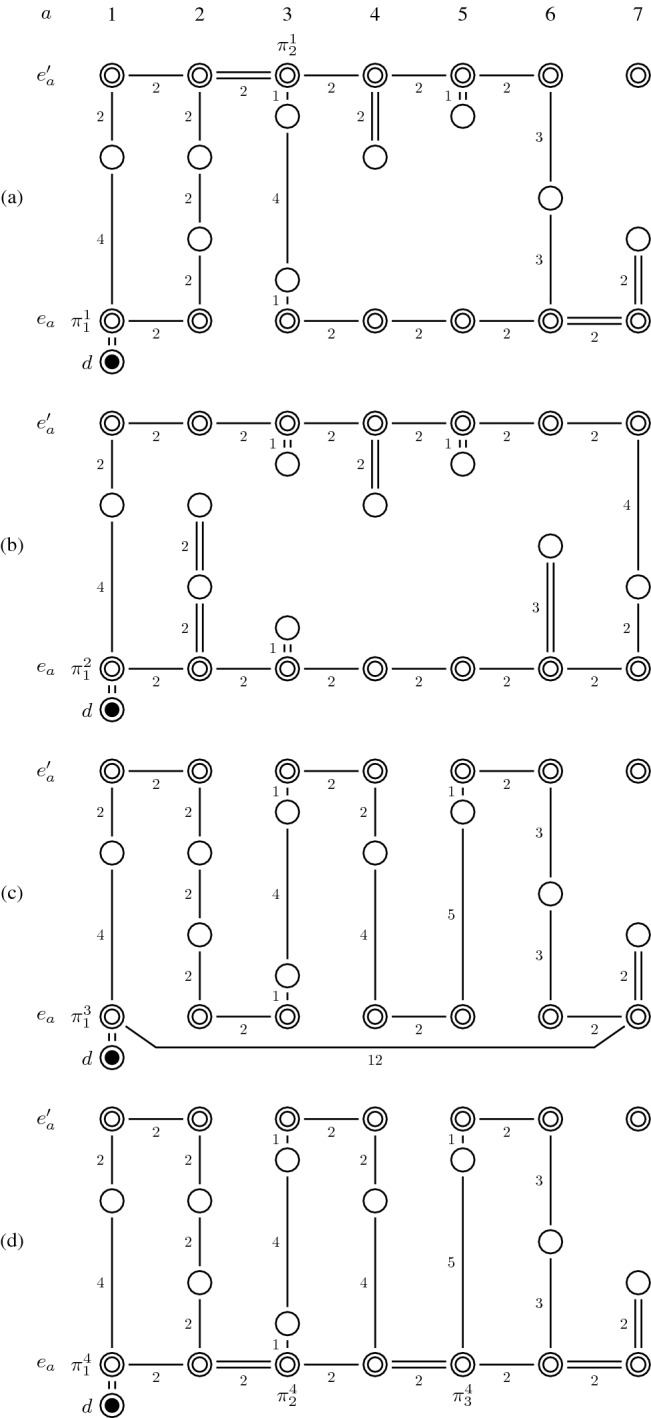
Example of tour subgraphs based on (a) optimal, (b) largest gap, (c) S-shape, and (d) S-shape+ routing policies

As described before, any routing policy can be used to generate a tour subgraph. In the numerical experiments, tour subgraphs are generated by the exact algorithm of Ratliff and Rosenthal (1983), which is called “optimal” routing policy in the following, the largest gap routing policy by Hall (1993), the S-shape routing policy by Goetschalckx and Ratliff (1988), and a newly introduced S-shape+ routing policy. Figure 7 illustrates the tour subgraphs that result from the optimal (a), the largest gap (b), the S-shape (c), and the S-shape+ (d) routing policy for a given picking order. For each tour subgraph, we indicate the degrees of freedom by $\pi ^k_j$. The routing policies are described as follows:

- *Largest gap:* According to the largest gap policy, the leftmost and the rightmost picking aisle are completely traversed if picks are required in there. The other picking aisles that contain a picking position are entered and left from the same cross aisle such that the largest gap is not traversed. A gap represents the distance between (i) any two adjacent picking positions, (ii) the first picking position and the front cross aisle, or (iii) the last picking position and the rear cross aisle. In the case of (i), a picking aisle is accessed via the front (or rear) cross aisle, whereas in the case of (ii) or (iii), a picking aisle is accessed from the rear (ii) or the front cross aisle (iii). The largest gap policy provides a single degree of freedom at the beginning of the tour.
- *S-shape:* In the S-shape routing policy, a picker enters picking aisles in an alternating manner from the front and the rear cross aisle and traverses them completely (if picks are required in there) or not at all (if no pick is required in there).
- *S-shape+:* As in the S-shape routing policy, in S-shape+, picking aisles are entered in an alternating manner from the front and the rear cross aisle and traversed completely (if picks are required). The difference between these two routing policies is the following: In the standard S-shape routing policy, the picker directly travels from the front location of the rightmost picking aisle to be visited to the depot without accessing the other front locations of the picking aisles (see Fig. 7(c)). Thus, all picks are executed on the way to the rightmost picking aisle that contains a picking position. In the S-Shape+ routing policy, the picker successively accesses each front location of the picking aisles on the way back to the depot. As shown in Fig. 7(d), this leads to a tour subgraph which allows the picker to skip required picking aisles on the way from the depot to the rightmost picking aisle to be visited and then to access the skipped picking aisles on the way back to the depot. Both routing policies lead to the same distance traveled by the picker but differ in the number of degrees of freedom they offer. The S-shape routing policy contains only one degree of freedom at the beginning of the tour, whereas the S-shape+ routing policy offers multiple degrees of freedom and therefore provides greater flexibility in designing the picker routes.

Combining the above described parameter values leads to 160 instance groups, which are identified by the demand scenario (UDD or CBD), the number of pickers |*K*|, the number of tour subgraphs per picker $|O^k|$, and the routing policy used to generate the respective tour subgraphs. For each instance group, we generate five instances, i.e., $160\!\cdot \! 5 = 800$ instances in total.

All experiments are conducted on a computing cluster running CentOS 7 with $2\times \hbox {Intel}$ Xeon E5-2430v2 Processors at 2.50 GHz and 64 GB of memory per compute node. Gurobi is used to solve the mixed integer program presented in Sect. 3.2, and each process runs multithreaded on 6 CPU cores. We restrict the solution time of Gurobi to 3600 s for all experiments. ILS is implemented in single-threaded C++ and compiled using GCC 8.2 with full optimizations enabled.

### Computational results

In this section, we assess the performance of the PTEP model and of our ILS. Moreover, we provide managerial insights with respect to the potential avoidance of temporal overlaps between pickers in the warehousing system under study.

Tables 2 and 3 present aggregate results for different routing policies on the UDD instances and Tables 4 and 5 on the CBD instances. Each table reports averages for groups of instances defined by the number of pickers (column *K*) and the number of tour subgraphs per picker (column $|O^{k}|$). The remaining columns are divided into two blocks, where each block reports the results for one of the underlying routing policies.

In column $f^{ rand }$, we give the average temporal overlap of 1000 randomly generated solutions, i.e., we randomly construct directed picking tours from the respective tour subgraphs. In addition, we report the average of the total travel distance of pickers (column $td$). All other values that are reported are provided as percentage deviation from the objective function value reported in column $f^{ rand }$. Column $ub$ (%) ($lb$ (%)) denotes the percentage deviation of the best objective function (lower bound) values given by Gurobi. Column $\#opt$ indicates the number of instances solved to proven optimality. For ILS, the reported results are based on five runs on each instance. In column $best$ (%) ($avg$ (%)), we show the percentage deviation of the best (average) solution value over the five runs. Column $t_{a}$ (*s*) gives the average runtimes in seconds.

In the following, we discuss the results of our experiments:

**Comparison of ILS to Gurobi** The results show that the number of pickers, the number of tour subgraphs per picker, and the routing policy significantly influence the performance of Gurobi. The main findings are as follows:

- *Optimal:* Using the optimal routing policy, Gurobi is able to solve 55 UDD instances and 74 CBD instances to optimality (UDD instances with |*K*| $=$ $\left\{ 2,5\right\}$ and those with |*K*| $=$ $\left\{ 10\right\}$ assuming $|O^{k}|$ $=$ $\left\{ 2,5\right\}$ as well as all CBD instances with |*K*| $=$ $\left\{ 2,5,10\right\}$). On the UDD and CBD instances with |*K*| $=$ 20, all instances with $|O^{k}|$ $=$ 2 are solved, and on the CBD instances with $|O^{k}|$ $=$ 5, four out of five instances are solved. On the instances with |*K*| $=$ 40, Gurobi is not able to solve any of the UDD and CBD instances within the given runtime limit.
- *Largest gap and S-shape:* Most of the instances can be solved to optimality using the largest gap policy (148 out of 200 instances), i.e., all UDD and CBD instances with |*K*| $=$ $\left\{ 2,5,10\right\}$, except a single CBD instance with |*K*| $=$ 10 and $|O^{k}|$ $=$ 20. On the CBD instances with |*K*| $=$ 20 and $|O^{k}|$ $=$ $\left\{ 2,5\right\}$, Gurobi provides optimal solutions for all tested instances and on the UDD instances, for all except for a single instance. Even on the larger UDD and CBD instances with |*K*| $=$ 40 and $|O^{k}|$ $=$ 2, Gurobi finds optimal solutions for all tested instances. Similar results can be observed for the case of the S-shape routing policy (146 out of 200 instances are solved to optimality).
- *S-shape+:* The fewest of the instances can be solved using the S-shape+ routing policy (106 out of 200 instances), i.e., all UDD and CBD instances with |*K*| $=$ $\left\{ 2,5\right\}$ and $|O^{k}|$ $=$ $\left\{ 2,5,10,20\right\}$. On the larger UDD instances, Gurobi finds optimal solutions for all instances with |*K*| $=$ 10 and $|O^{k}|$ $=$ 2, and for 4 out of 5 instances with |*K*| $=$ 20 and $|O^{k}|$ $=$ 2. In the case of larger CBD instances, Gurobi provides optimal solutions for all instances with |*K*| $=$ $\left\{ 10,20\right\}$ and $|O^{k}|$ $=$ 2, and for 4 out of 5 instances with |*K*| $=$ 10 and $|O^{k}|$ $=$ 5, and 3 out of 5 instances with |*K*| $=$ 40 and $|O^{k}|$ $=$ 2.

The results show that Gurobi succeeds in solving small and medium instances within reasonable runtimes but is not able to consistently solve larger instances to optimality within the given time limit. The tables show that ILS finds the same or slightly worse solutions as Gurobi on the smaller and medium instances but clearly outperforms Gurobi on the larger instances: It is worth noting that the solution quality of ILS on the largest instances is close to the lower bound of Gurobi (in the cases in which the lower bound is not equal to zero). In addition, the results indicate a very good robustness of our ILS, i.e., the best solutions found by ILS deviate from the average solutions by only 0.5% for the UDD case and 0.6% for the CBD case. The runtimes of ILS are reasonable and stay below 10 minutes for all but the largest instances with |*K*| $=$ 40 and $|O^{k}|$ $=$ 20. The results of ILS clearly demonstrate that ILS is able to solve the largest instances within reasonable runtimes.

**Table 2:**
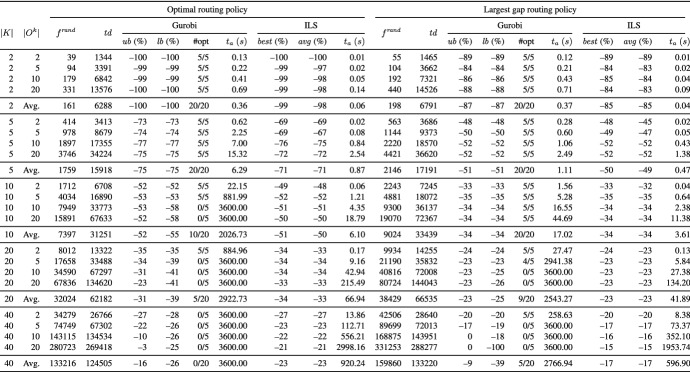
Results on the UDD instances grouped by number of pickers |*K*| and number of tour subgraphs per picker $|O^{k}|$ using the optimal and largest gap routing policy

**Table 3:**
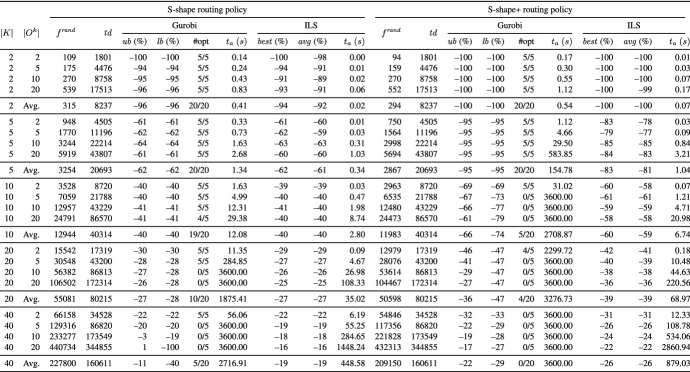
Results on the UDD instances grouped by number of pickers |*K*| and number of tour subgraphs per picker $|O^{k}|$ using the S-shape and S-shape+ routing policy

**Table 4:**
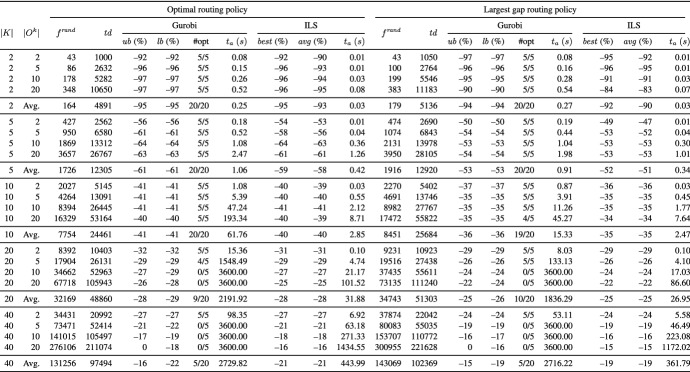
Results on the CBD instances grouped by number of pickers |*K*| and number of tour subgraphs per picker $|O^{k}|$ using the optimal and largest gap routing policy

**Table 5:**
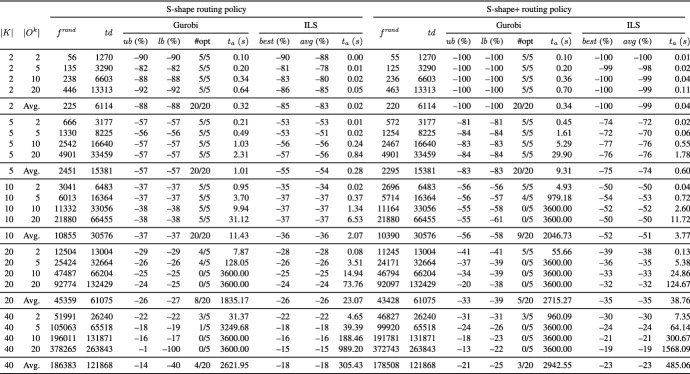
Results on the CBD instances grouped by number of pickers |*K*| and number of tour subgraphs per picker $|O^{k}|$ using the S-shape and S-shape+ routing policy

**Performance of ILS for different routing policies** In the following, we investigate the performance of ILS for different routing policies. To this end, we compare the results of the arbitrary solution (see column “$f^{ rand }$”) with those obtained by our ILS (see column “$best$”) for the same picker routing policy. We use the reduction in temporal overlap between pickers to compare the results of an arbitrary solution to the PTEP with that obtained by our ILS for the same picker routing policy. This can be an insightful measure for managers if a fixed routing policy is used in their warehouse.

The results show that ILS is able to significantly reduce the temporal overlap between pickers independent of the picker routing policy at hand. For example, on the smallest UDD and CBD instances with |*K*| $=$ 2 and S-shape+ routing policy, ILS generates solutions that are completely free of temporal overlaps. Even on the largest UDD (CBD) instances with |*K*| $=$ $\left\{ 20,40\right\}$, a reduction of 39% (35%) and 26% (23%) is achieved although the instances assume a warehouse with only 10 picking aisles. ILS achieves the smallest reduction in the temporal overlap between pickers in the case of S-shape and largest gap routing policies. For instance, even on the smallest UDD instances with |*K*| $=$ 2, temporal overlaps between pickers cannot be completely avoided. This is because both routing policies provide only one degree of freedom in the routing: the picker performs the S-shape routing either via the leftmost or via the rightmost picking aisle to be visited, and the largest gap routing starts either along the front or the rear cross aisle.

In the following, we investigate the effect of (i) the number of pickers, (ii) the number of tour subgraphs per picker, and (iii) the demand scenario on the total temporal overlap between pickers. Moreover, we briefly discuss the results with respect to the total travel distance of the pickers.

- For all routing policies, we observe that the larger the number of pickers |*K*|, the smaller the average reduction in the total temporal overlap between pickers. Obviously, in the case of a large number of pickers, there is less potential for reduction in comparison with a small number of pickers because the probability of temporal overlaps increases with the number of pickers. For instance, on the UDD instances using the S-shape+ routing policy, the average reduction ranges between 26% in the case of |*K*| $=$ 40 and 100% for |*K*| $=$ 2.
- The influence of the number of tour subgraphs per picker is hardly visible. For example, on the UDD instances with |*K*| $=$ 20 using largest gap routing policy, the reductions achieved for $|O^{k}|$ $=$ $\left\{ 2,5,10,20\right\}$ are 24%, 23%, 23%, and 23%, respectively.
- For CBD, ILS generates solutions that are slightly worse compared to those for UDD. For example, for CBD, |*K*| $=$ 40, and the S-shape+ routing policy, ILS yields a reduction of 23% on average compared to 26% for UDD.
- As expected, the optimal routing leads to the shortest picking tours, and the S-shape and S-shape+ routing policies result in the longest routes. This is because all picking aisles containing even a single item to be picked must be completely traversed.

Our numerical results show that large reductions in the total temporal overlap between pickers are achieved independent of the underlying routing policy. The smallest temporal overlap is observed for the optimal routing policy (see Tables 7, 8, 9, and 10). With respect to picking performance, the optimal routing policy is obviously superior to the other routing policies because it leads to shorter picking tours. However, a shortcoming of using the optimal routing policy is that pickers may get confused by the complex routes, and therefore, tend to deviate from optimal routing patterns (see, e.g., Elbert et al. 2017). Deviating from given routes can have a negative impact on picking efficiency and also increase the risk of infection spread. This effect is particularly strong if no modern technologies such as tablets, pick-by-light, or pick-by-voice are used to assist pickers.

To reduce infection risk in picker-to-parts warehousing systems through our PTEP, it is paramount that pickers follow prescribed routes. While pickers will not always follow prescribed routes in practice in non-pandemic periods for several reasons (e.g., social interactions), in pandemic periods, pickers are more likely to do so to avoid infection. Moreover, although our PTEP is not able to completely prevent possibilities for social interaction, it significantly reduces them through exploiting the degrees of freedom that the routing policies offer.

## Zone picking

**Fig. 8:**
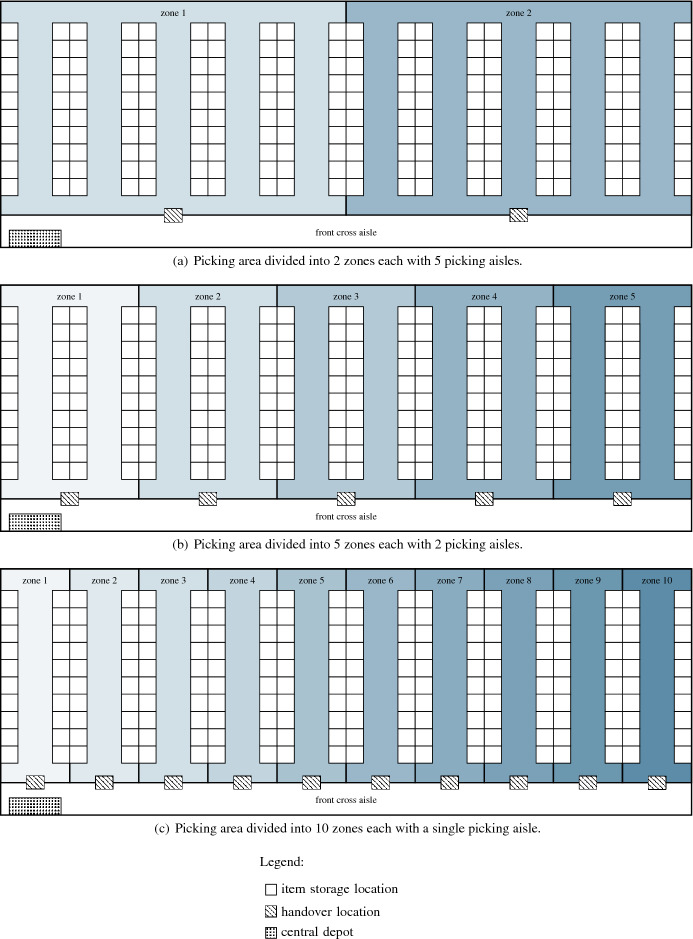
Warehouse layout assumed in the zone picking system

In this section, we compare our approach to a zone picking approach, which is another possible way to reduce infection risk in picker-to-parts warehouses. To make a fair comparison possible, we choose the following setup for the zone picking approach:

- *Warehouse layout:* The picking area follows the single-block parallel-aisle warehouse layout described in Sect. 5.1, but is divided into smaller zones, each with a single picker assigned to it. A zone comprises a fixed number of picking aisles, and each zone is associated with a handover location located in the front cross aisle. Figure 8 illustrates the zone picking system with different numbers of zones. Obviously, physical distancing practices are easy to implement, and infection risk between pickers can be almost eliminated in such systems.
- *Parallel zone picking:* We assume parallel zoning, i.e., the items of a picking order are simultaneously retrieved by multiple zone pickers in multiple zones as described in the following: Each zone picker is initially positioned at her handover location. Starting from the handover location, the zone pickers collect the picking order items from their zone according to an optimal routing pattern (see Ratliff and Rosenthal 1983). Once a zone picker has collected all picking order items from her zone, she returns to her handover location to deposit the items. Subsequently, she executes the next picking order from her picking list, starting from her handover location. We assume that each zone picker uses a bin with sufficient capacity for temporarily storing the picking order items and that each handover location has infinite storage capacity (buffer) for depositing items.
- *Sequence for picking orders:* The sequence according to which the picking orders have to be collected by the zone pickers is given.
- *Consolidator:* A consolidator collects the items of a single picking order from the relevant handover locations (i.e., those from which the picking order items are to be collected) on a single tour as follows: The consolidator starts at the depot, where she is equipped with a trolley with sufficient capacity for the items of a single picking order, proceeds along the front cross aisle to the relevant handover locations, and from the last visited handover location to the depot. The sequence according to which a consolidator visits the relevant handover locations is determined in optimal fashion (concerning the objective of minimizing the completion time of a picking order). We assume that no effort for consolidating the items occurs at the depot. The consolidator collects the picking orders in the given picking sequence.
- *Test instances:* Our experiment is based on the instances described in Sect. 5.1. For both approaches, we use the exact algorithm of Ratliff and Rosenthal (1983) to determine a picking tour. For our PTEP, we consider |*K*| $=$ $\left\{ 3, 6, 11\right\}$ pickers and $|O^k|$ $=$ $\left\{ 2, 5, 10, 20\right\}$ tour subgraphs per picker *k* $\in$ *K*. For the zoning approach, the picking area is divided into 2, 5, and 10 zones comprising 5, 2, and 1 picking aisles, respectively. We assume |*K*| $=$ $\left\{ 3, 6, 11\right\}$ pickers, where the 3rd, 6th, and 11th picker represents the consolidator. The number of picking orders in the zone picking system is defined as |*O*| $=$ $|O^k|$ $\cdot$ |*K*|. Combing the above described parameter values leads to 12 instance groups, which are identified by the number of pickers |*K*| and the number of picking orders in the system |*O*|. For each instance group, we generate 5 instances, i.e., 12 $\cdot$ 5 $=$ 60 instances in total.

In the following, we use the makespan, i.e., the time when all picking orders are completed, as performance measure for comparing the PTEP and the zone picking approach. In Table 6, we present aggregate results obtained by our ILS for the PTEP and by the zone picking approach on the UDD instances and the CBD instances. The table reports averages for groups of instances defined by the number of pickers (column |*K*|) and the number of picking orders in the system (column |*O*|). The remaining columns are divided into two blocks, where the first block represents the results on the UDD instances and the second block those on the CBD instances. In column *ATO*, we give the average temporal overlap. Column $M_{PTEP}$ ($M_{ZONE}$) reports the average makespan required for the PTEP (zone picking approach). Column $\Delta \,(\%)$ denotes the average percentage deviation between $M_{ZONE}$ and $M_{PTEP}$.

**Table 6:**
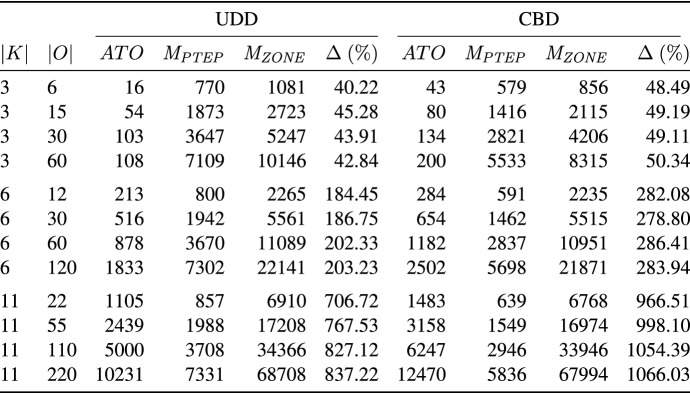
Results on the UDD and CBD instances

The results show that our PTEP approach strongly outperforms the zone picking approach with respect to makespan. For example, on the smallest instances with |*K*| $=$ 3 pickers and |*O*| $=$ 6 picking orders in the system, the average percentage deviation between the PTEP and the zone picking approach is approximately 40% in the UDD case (49% in the CBD case). Moreover, on these instances, our ILS finds solutions that are almost free of temporal overlaps between pickers. The larger the number of pickers |*K*|, the greater the deviation in makespan between the PTEP and the zone picking approach. For example, on the largest CBD instances with |*K*| $=$ 11 pickers and |*O*| $=$ 220 picking orders in the system, the makespan obtained by the zone picking approach is on average 1066% higher than that obtained by our ILS for the PTEP.

To sum up, the zone picking approach presented above significantly increases the makespan although many favorable assumptions are made (e.g., no effort for consolidating the collected items at the depot, infinite storage capacity for depositing items at the handover locations). Clearly, the zone picking approach is able to prevent pickers from operating in close proximity to each other, and thus, to almost eliminate the infection risk between pickers. Consequently, the tradeoff between basically no temporal overlap but excessive makespan (zone picking approach) and some temporal overlap without raising costs (PTEP) must be evaluated by warehouse managers. Given medical tests and personal protective measures (e.g., vaccinations and/or mouth-nose protection masks), we believe that our PTEP is an interesting approach for warehouse managers to reduce infection risk between pickers without compromising order picking performance.

## Conclusion

This paper aims to reduce the risk of infection in a picker-to-parts warehousing system in which multiple pickers operate in the same picking area. To this end, we introduce the PTEP that generates directed picking tours from given tour subgraphs such that the time period in which pickers simultaneously occupy the same picking aisles is minimized. We formally describe the PTEP as a mixed integer program and provide an efficient ILS.

Our main finding is that significant reductions in the total temporal overlap between pickers can be achieved by exploiting the degrees of freedom that routing policies offer. On average, a reduction in approximately 52% is achieved in the UDD scenario, while the reduction in the CBD scenario is slightly lower with 49%. The smallest temporal overlap between pickers is achieved in the case of the optimal routing policy. The S-shape routing policy tends to perform worst.

We believe that our results are quite interesting for warehouse managers in times of pandemics: Avoiding temporal overlaps between pickers does not only reduce the infection risk, but also increases the order picking efficiency (e.g., by reducing in-aisle congestion). Moreover, the reductions are achieved without compromising order picking performance, i.e., without changing the distance traveled (or time required) by the pickers. The comparison of our approach to a zone picking approach reveals that the zone picking approach results in excessive makespan: the deviation between the PTEP and the zone picking approach amounts to 396% on average.

To further reduce temporal overlaps between pickers, our problem could be extended to incorporate modern warehousing concepts, such as scattered storage or multiple end depots. For example, with scattered storage, an item type can be available from several storage locations (see Goeke and Schneider 2018). Assigning the stock keeping units of a frequently requested item type to multiple storage locations could reduce the probability of pickers working close to each other. The PTEP could be extended to incorporate congestion in picking aisles and picker blocking, which is particularly relevant for warehouses with narrow picking aisles. Moreover, future research could investigate alternative objective functions for reducing the infection risk, e.g., one could minimize infection risk that results from pickers operating within a given physical distance for more than a critical period of time.

Pandemics or epidemics will certainly be given more focus in future research. Incorporating infection risks into well-known order picking problems will pose new challenges in the research on warehousing to ensure a safe order picking environment during pandemics.

## Figures and Tables

**Table 7:**
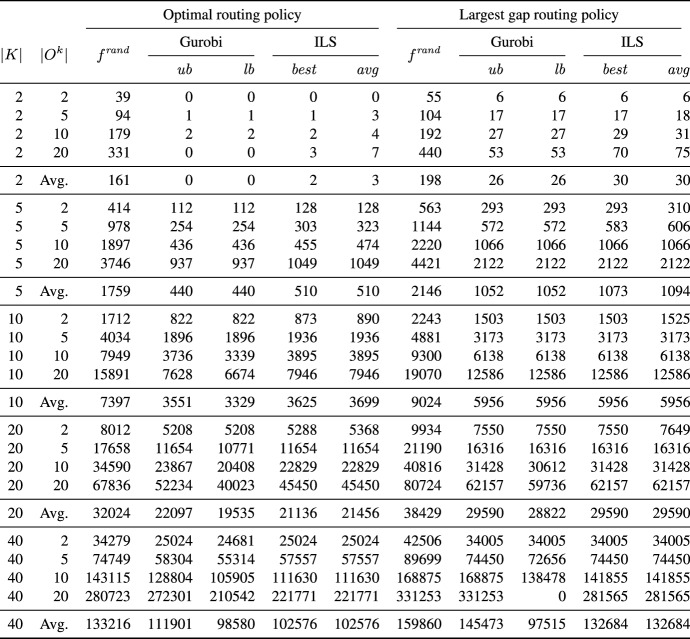
Results on the UDD instances grouped by number of pickers |*K*| and number of tour subgraphs per picker $|O^{k}|$ using the optimal and largest gap routing policy

**Table 8:**
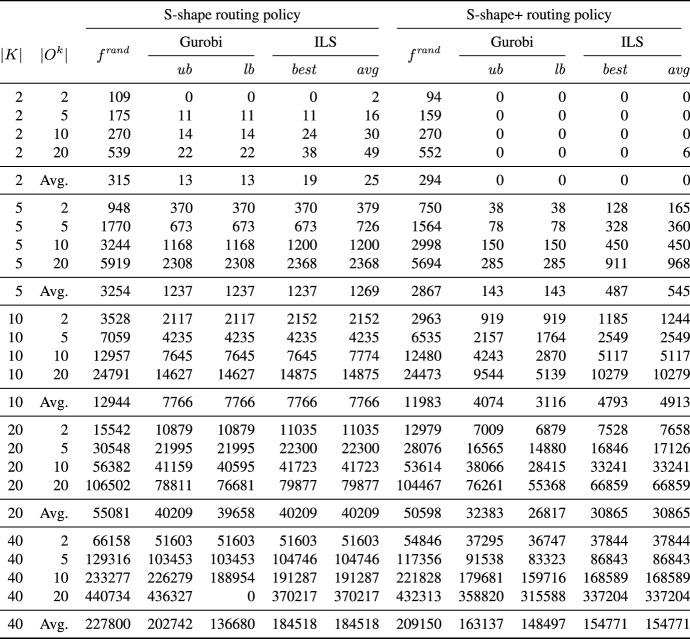
Results on the UDD instances grouped by number of pickers |*K*| and number of tour subgraphs per picker $|O^{k}|$ using the S-shape and S-shape+ routing policy

**Table 9:**
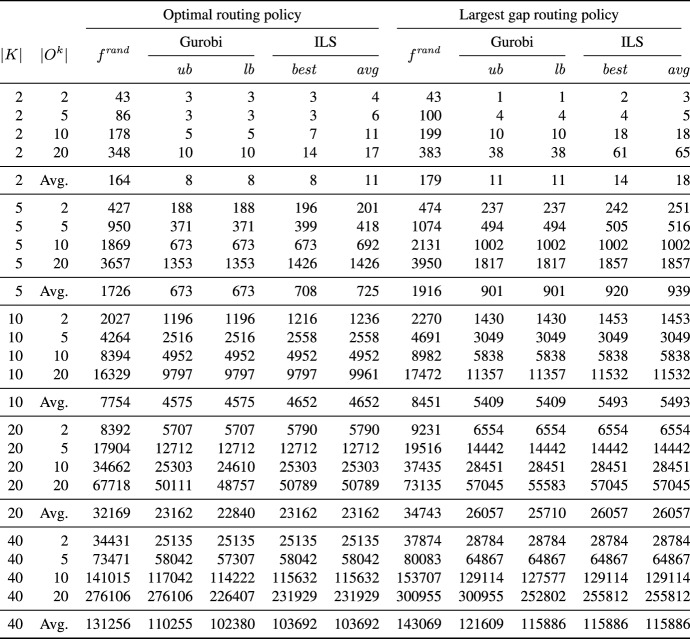
Results on the CBD instances grouped by number of pickers |*K*| and number of tour subgraphs per picker $|O^{k}|$ using the optimal and largest gap routing policy

**Table 10:**
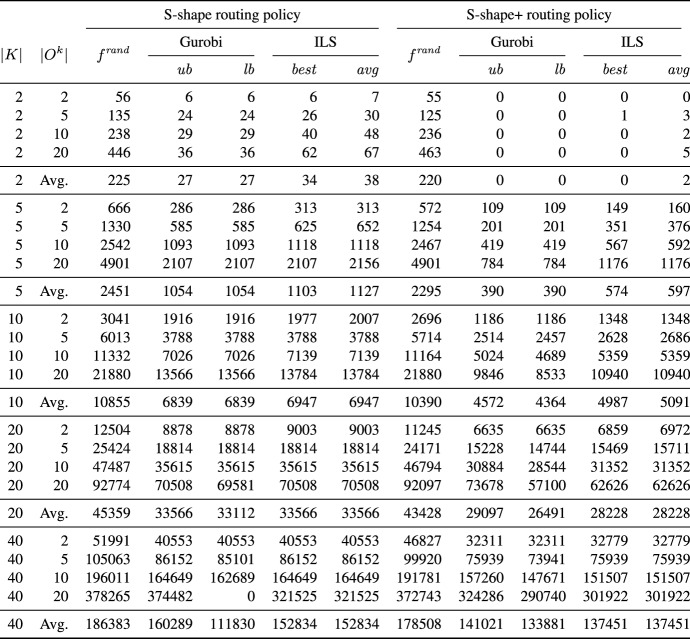
Results on the CBD instances grouped by number of pickers |*K*| and number of tour subgraphs per picker $|O^{k}|$ using the S-shape and S-shape+ routing policy

## Data Availability

The data that support the findings of this study are available from the corresponding author upon request.

## Appendix: Additional results

Tables 7 and 8 present aggregate results for different routing policies on the UDD instances and Tables 9 and 10 on the CBD instances. Each table reports averages for groups of instances defined by the number of pickers (column *K*) and the number of tour subgraphs per picker (column $|O^{k}|$). The remaining columns are divided into two blocks, where each block reports the results for one of the underlying routing policies.

In column $f^{ rand }$, we give the average temporal overlap of 1000 randomly generated solutions, i.e., we randomly construct directed picking tours from the respective tour subgraphs. Column $ub$ ($lb$) indicates the average temporal overlap of the best objective function (lower bound) values given by Gurobi. For ILS, the reported results are based on five runs on each instance. In column $best$ ($avg$), we show the average temporal overlap of the best (average) solution value over the five runs.

## Footnotes

1 For the sake of readability, we have decided to speak exclusively of female pickers. Of course, a picker can also be of any other gender.

## References

[CR1] Amazon (2020) The company’s latest innovation provides real-time social distancing feedback and we plan to open source the technology, 2020. Available online at https://www.aboutamazon.com/news/operations/amazon-introduces-distance-assistant. Accessed 8 Feb 2022

[CR2] Ardjmand E, Singh M, Shakeri H, Tavasoli A, Young WA (2021). Mitigating the risk of infection spread in manual order picking operations: a multi-objective approach. Appl Soft Comput.

[CR3] Azadeh K, de Koster RBM, Roy D (2019). Robotized and automated warehouse systems: review and recent developments. Transp Sci.

[CR4] Boysen N, de Koster RBM, Weidinger F (2019). Warehousing in the e-commerce era: a survey. Eur J Oper Res.

[CR5] Boysen N, de Koster RBM, Füßler D (2021). The forgotten sons: warehousing systems for brick-and-mortar retail chains. Eur J Oper Res.

[CR6] Chen F, Wang H, Qi C, Xie Y (2013). An ant colony optimization routing algorithm for two order pickers with congestion consideration. Comput Ind Eng.

[CR7] Chen F, Wang H, Xie Y, Qi C (2014). An ACO-based online routing method for multiple order pickers with congestion consideration in warehouse. J Intell Manuf.

[CR8] DC Velocity (2020) In pandemic times, DC managers rethink their labor strategies, 2020. Available online at https://www.dcvelocity.com/articles/48024-in-pandemic-times-dc-managers-rethink-their-labor-strategies. Accessed 8 Feb 2022

[CR9] de Koster RBM, Le-Duc T, Roodbergen KJ (2007). Design and control of warehouse order picking: a literature review. Eur J Oper Res.

[CR10] Elbert RM, Franzke T, Glock CH, Grosse EH (2017). The effects of human behavior on the efficiency of routing policies in order picking: the case of route deviations. Comput Ind Eng.

[CR11] Goeke D, Schneider M (2018) Modeling single picker routing problems in classical and modern warehouses. Working paper, DPO-2018-11 (version 1, 04.11.2018), Deutsche Post Chair—Optimization of Distribution Networks, RWTH Aachen University

[CR12] Goetschalckx M, Ratliff HD (1988). Order picking in an aisle. IIE Trans.

[CR13] Grosse EH, Glock CH, Ballester-Ripoll R (2014). A simulated annealing approach for the joint order batching and order picker routing problem with weight restrictions. Int J Oper Quant Manag.

[CR14] Gue KR, Meller RD, Skufca JD (2006). The effects of pick density on order picking areas with narrow aisles. IIE Trans.

[CR15] Hall RW (1993). Distance approximations for routing manual pickers in a warehouse. IIE Trans.

[CR16] Henn S, Wäscher G (2012). Tabu search heuristics for the order batching problem in manual order picking systems. Eur J Oper Res.

[CR17] Hong S, Johnson AL, Peters BA (2012). Batch picking in narrow-aisle order picking systems with consideration for picker blocking. Eur J Oper Res.

[CR18] Masae M, Glock CH, Grosse EH (2019). Order picker routing in warehouses: a systematic literature review. Int J Prod Econ.

[CR19] Napolitano M (2012). 2012 warehouse/DC operations survey: mixed signals. Mod Mater Handl.

[CR20] Pan JC-H, Shih P-H (2008). Evaluation of the throughput of a multiple-picker order picking system with congestion consideration. Comput Ind Eng.

[CR21] Parikh PJ, Meller RD (2009). Estimating picker blocking in wide-aisle order picking systems. IIE Trans.

[CR22] Ratliff HD, Rosenthal AS (1983). Order picking in a rectangular warehouse: a solvable case of the traveling salesman problem. Oper Res.

[CR23] Schrotenboer AH, Wruck S, Roodbergen KJ, Veenstra M, Dijkstra AS (2017). Order picker routing with product returns and interaction delays. Int J Prod Res.

[CR24] Statista (2020) Total retail sales worldwide from 2017 to 2023, 2019. Available online at https://www.statista.com/statistics/443522/global-retail-sales. Accessed 1 Mar 2021

[CR25] Tompkins JA, White JA, Bozer YA, Tanchoco JMA (2010) Facilities planning, 4th edn. Wiley

[CR26] Weidinger F (2018). Picker routing in rectangular mixed shelves warehouses. Comput Oper Res.

[CR27] World Health Organization. Transmission of SARS-CoV-2, 2020. Available online at https://www.https://www.who.int/news-room/commentaries/detail/transmission-of-sars-cov-2-implications-for-infection-prevention-precautions. Accessed 6 April 2021

[CR28] Zhang M, Batta R, Nagi R (2009). Modeling of workflow congestion and optimization of flow routing in a manufacturing/warehouse facility. Manage Sci.

